# Prognosis of Extracapsular Spread of Cervical Lymph Node Metastases in Nasopharyngeal Carcinoma

**DOI:** 10.3389/fonc.2020.523956

**Published:** 2020-09-25

**Authors:** Xue Yin, Lu Lv, Xin-Bin Pan

**Affiliations:** ^1^Department of Oncology, The Central Hospital of Wuhan, Tongji Medical College, Huazhong University of Science and Technology, Wuhan, China; ^2^Department of Radiation Oncology, Guangxi Medical University Cancer Hospital, Nanning, China

**Keywords:** nasopharyngeal carcinoma, extracapsular spread, NPC, ECS, prognosis

## Abstract

**Purpose:**

This study aims to identify the prognosis of the extracapsular spread (ECS) of cervical lymph node metastases in nasopharyngeal carcinoma (NPC).

**Materials and Methods:**

Patients with NPC were extracted from the Surveillance, Epidemiology, and End Results (SEER) database from 2004 to 2016. Pathologically confirmed World Health Patients with World Health Organization types I, II, and III NPC with complete ECS data of cervical lymph node metastases were investigated. The included patients were divided into non-ECS and ECS groups. The 10-year overall survival (OS) and cancer-specific survival (CSS) were compared between the two groups using the Kaplan-Meier method and propensity score matching analyses.

**Results:**

A total of 625 patients were included. The ECS group included 99 (15.84%) patients. The non-ECS group included 526 (84.16%) patients. The 10-year OS (50.2 vs. 35.8%; *P* < 0.001) and CSS (64.8 vs. 45.7%; *P* < 0.001) were better in the non-ECS group than in the ECS group in the unmatched cohort. Propensity score matching analyses revealed favorable 10-year OS (52.7 vs. 35.8%; *P* = 0.008) and CSS (61.2 vs. 45.7%; *P* = 0.008) in the non-ECS group with respect to the ECS group. Age, sex, race, AJCC stage, and ECS (hazard ratio (HR) = 1.71, 95% confidence interval (CI), 1.14–2.57, *P* = 0.010) were independent prognostic factors for OS. Age, sex, AJCC stage, and ECS (HR = 1.91; 95% CI, 1.21–3.01; *P* = 0.005) were independent prognostic factors for CSS.

**Conclusion:**

This study indicated that ECS is a prognostic risk factor for NPC. Further studies should be performed to verify the results due to the limitations of the SEER database.

## Introduction

Nasopharyngeal carcinoma (NPC) is a highly epidemiological cancer in South China (1, 2). It is a unique head and neck cancer with a high incidence of cervical lymph node metastases. Approximately 85% of patients present with cervical lymph node metastases at diagnosis (3). Moreover, extracapsular spread (ECS) has been found in 33.6–75.6% of patients with cervical lymph node metastases (4–6). In other head and neck cancers, ECS has been suggested to be a risk prognostic factor (7, 8). However, the prognosis of the ECS of cervical lymph node metastases in NPC has not been well investigated. Mao et al. (4) revealed that ECS was an independent risk prognostic factor. In contrast, several studies reported that ECS was not associated with survival (5, 6, 9). To identify the prognosis of patient with NPC with ECS of cervical lymph node metastases, this retrospective cohort study was conducted using data from the Surveillance, Epidemiology, and End Results (SEER) database.

## Materials and Methods

### Patients

Patients with NPC from the SEER database between 2004 and 2016 were investigated. The inclusion criteria were as follows: (1) patients with pathologically confirmed NPC; (2) patients with World Health Organization (WHO) type I, II, or III NPC; (3) patients with definite American Joint Committee on Cancer (AJCC) TNM stages; (4) patients with a definite tumor grade; and (5) patients with definite information on the ECS of cervical lymph node metastases. Included patients were divided into ECS and non-ECS groups.

According to the AJCC, ECS is assessed by physical examination and imaging [computed tomography (CT) scan or magnetic resonance imaging (MRI)] (10). ECS can be diagnosed clinically by a matted mass of nodes adherent to overlying skin or adjacent soft tissue or clinical evidence of cranial nerve invasion. The criteria of radiological signs of ECS were as follows: (1) amorphous and speculated margins of a metastatic node; (2) the presence of indistinct nodal margins, irregular nodal capsular enhancement, or infiltration into the adjacent fat or muscle; and (3) fusion status of metastatic nodes (11–13). In the SEER database, ECS was coded as “001,” while non-ECS was coded as “000” under the variable “CS Site-Specific Factor 2.”

### Endpoints

The primary endpoint was overall survival (OS). OS was defined in the SEER database as the time from diagnosis to death as a result of any cause. The secondary endpoint was cancer-specific survival (CSS). CSS was defined as the time from diagnosis to death attributed to NPC.

### Statistical Analysis

The continuous variable of age was transformed into a categorical variable (14). Categorical variables, including age, sex, race, tumor grade, tumor pathology, AJCC stage, T stage, N stage, M stage, radiotherapy, and chemotherapy, between the non-ECS and ECS groups were analyzed by using the χ^2^ test or Fisher’s exact test. Logistic regression analysis was performed to identify factors associated with ECS.

The 10-year OS and CSS rates of the ECS and non-ECS groups were calculated using Kaplan-Meier analysis. The survival difference between the ECS and non-ECS groups was examined by the log-rank test. Multivariable proportional hazards models adjusted for age, sex, race, tumor grade, tumor pathology, AJCC stage, radiotherapy, and chemotherapy were implemented to assess independent prognostic factors. The results are reported as hazard ratios (HRs) with 95% confidence intervals (CIs).

A matched case-control analysis was conducted by using propensity score matching (PSM) to reduce the influence of selection bias on the comparison between ECS and non-ECS groups. A logistic regression model was established in which ECS status was taken as the dependent variable in the process of calculating the propensity scores. One-to-one matching without replacement was performed using the nearest-neighbor match on the logit of the propensity score for confounding factors (derived from age, sex, race, tumor grade, tumor pathology, AJCC stage, radiotherapy, and chemotherapy). Standard differences for each of the covariates were used to compare the similarity of ECS and non-ECS groups after matching. An absolute value < 0.05 indicates that covariates are well balanced in both groups (15).

SPSS Statistics Version 26.0 software (IBM Co., Armonk, NY, United States) and R software (version 3.6.2) were used to perform statistical analyses. Two-tailed *P* < 0.05 was considered statistically significant.

## Results

### Patient Characteristics

**FIGURE 1:**
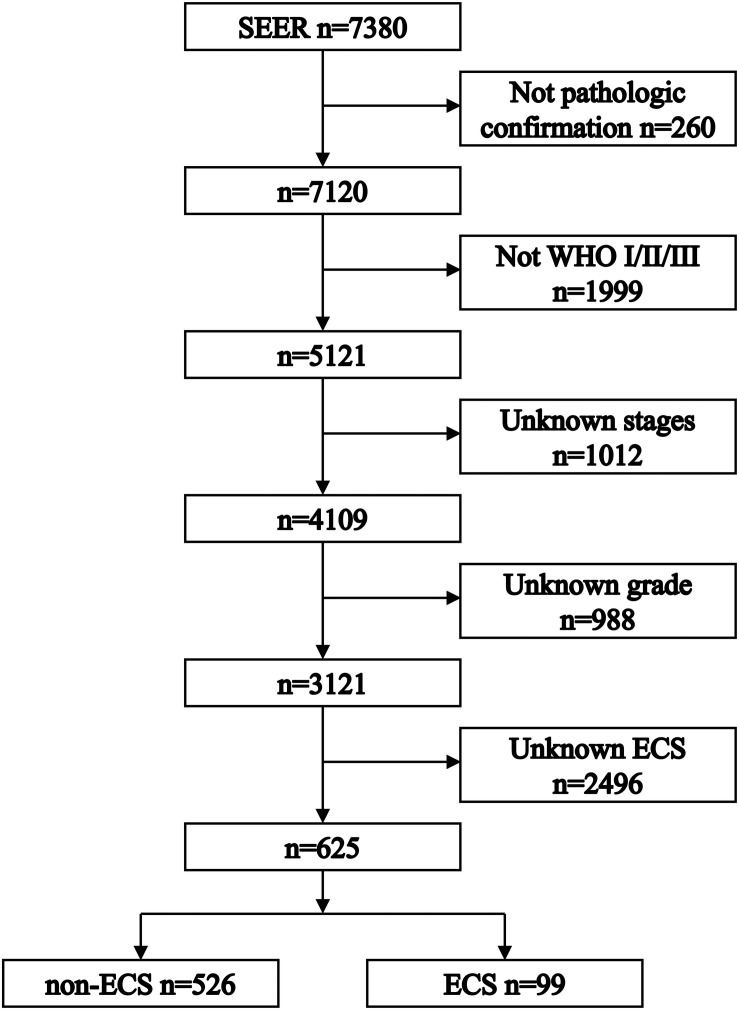
Flowchart depicting patient selection. ECS, extracapsular spread.

Figure 1 shows the process of patient selection. This study finally included 625 patients. The ECS group included 99 (15.84%) patients. The non-ECS group included 526 (84.16%) patients. Table 1 shows the patient characteristics. The median follow-up times were 85 months (interquartile range (IQR), 24–113), 45 months (IQR, 8–109), and 88 months (IQR, 30–114) for the whole group, ECS group, and non-ECS group, respectively. All patients were diagnosed before 2010. Consequently, the 6th edition AJCC staging system was applied to the patients in this study.

**TABLE 1:** Patient characteristics.

	Total (*n* = 625)	Non-ECS (*n* = 526)	ECS (*n* = 99)	*P* value
**Age**				
≤19	23 (3.68%)	18 (3.42%)	5 (5.05%)	Reference
20–39	90 (14.40%)	76 (14.45%)	14 (14.14%)	0.497
40–59	326 (52.16%)	285 (54.18%)	41 (41.41%)	0.209
60–79	171 (27.36%)	135 (25.67%)	36 (36.36%)	0.940
≥80	15 (2.40%)	12 (2.28%)	3 (3.03%)	0.898
**Sex**				
Female	165 (26.40%)	147 (27.95%)	18 (18.18%)	0.043
Male	460 (73.60%)	379 (72.05%)	81 (81.82%)	
**Race**				
Asian	238 (38.08%)	207 (39.35%)	31 (31.31%)	Reference
Black	74 (11.84%)	59 (11.22%)	15 (15.15%)	0.125
White	313 (50.08%)	260 (49.43%)	53 (53.54%)	0.206
**Grade**				
I	17 (2.72%)	14 (2.66%)	3 (3.03%)	Reference
II	80 (12.80%)	64 (12.17%)	16 (16.16%)	0.824
III	269 (43.04%)	226 (42.97%)	43 (43.43%)	0.856
IV	259 (41.44%)	222 (42.21%)	37 (37.37%)	0.703
**Pathology**				
WHO I	267 (42.72%)	218 (41.44%)	49 (49.49%)	Reference
WHO II	160 (25.60%)	135 (25.67%)	25 (25.25%)	0.471
WHO III	198 (31.68%)	173 (32.89%)	25 (25.25%)	0.095
**AJCC stage**				
II	190 (30.40%)	171 (32.51%)	19 (19.19%)	Reference
III	234 (37.44%)	206 (39.16%)	28 (28.28%)	0.521
IVa	58 (9.28%)	47 (8.94%)	11 (11.11%)	0.067
IVb	79 (12.64%)	58 (11.03%)	21 (21.21%)	0.001
IVc	64 (10.24%)	44 (8.37%)	20 (20.20%)	<0.001
**T stage**				
T1	225 (36.00%)	190 (36.12%)	35 (35.35%)	Reference
T2	182 (29.12%)	163 (30.99%)	19 (19.19%)	0.130
T3	129 (20.64%)	108 (20.53%)	21 (21.21%)	0.858
T4	89 (14.24%)	65 (12.36%)	24 (24.24%)	0.020
**N stage**				
N1	298 (47.68%)	263 (50.00%)	35 (35.35%)	Reference
N2	226 (36.16%)	193 (36.69%)	33 (33.33%)	0.335
N3	101 (16.16%)	70 (13.31%)	31 (31.31%)	<0.001
**M stage**				
M0	561 (89.76%)	482 (91.63%)	79 (79.80%)	<0.001
M1	64 (10.24%)	44 (8.37%)	20 (20.20%)	
**Radiotherapy**				
No	62 (9.92%)	46 (8.75%)	16 (16.16%)	0.024
Yes	563 (90.08%)	480 (91.25%)	83 (83.84%)	
**Chemotherapy**				
No	61 (9.76%)	49 (9.32%)	12 (12.12%)	0.388
Yes	564 (90.24%)	477 (90.68%)	87 (87.88%)	

*ECS, extracapsular spread; WHO, World Health Organization; AJCC, the American Joint Committee on Cancer.*

### Associated Factors of ECS

**FIGURE 2:**
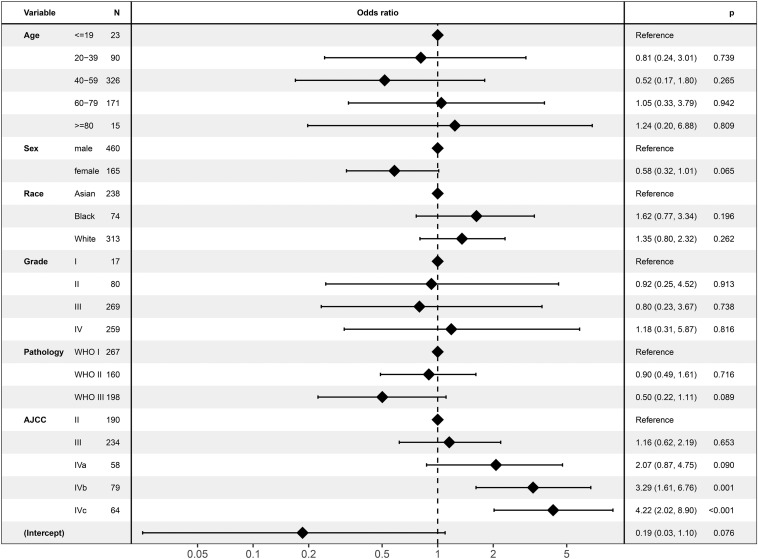
Logistic regression analysis for associated variables of extracapsular spread.

In the logistic regression analysis, ECS was only associated with AJCC stage (Figure 2). ECS was more likely to present in stage IVb [odds ratio (OR) = 3.29; 95% CI, 1.61–6.67; *P* = 0.001] and stage IVc (OR = 4.22; 95% CI, 2.02–8.90; *P* < 0.001) than in stage II NPC. However, ECS status was not different between stages II, III, and IVa. ECS was not correlated with age, sex, race, tumor grade, or pathology.

### Survival Before PSM

**FIGURE 3:**
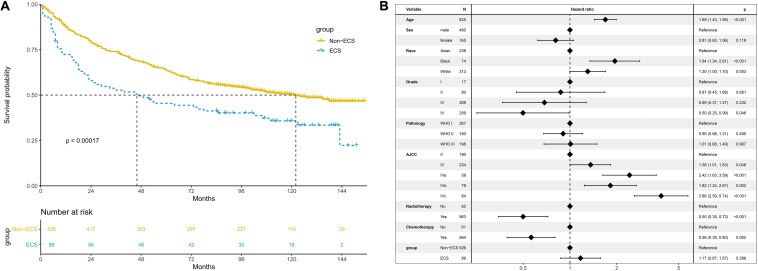
Prognosis of ECS for OS in the unmatched cohort. **(A)** OS between the ECS and non-ECS groups. **(B)** Multivariable regression analysis of prognostic factors for OS. ECS, extracapsular spread; OS, overall survival.

The 10-year OS of the non-ECS group was better than that of the ECS group (50.2 vs. 35.8%, *P* < 0.001; Figure 3A). However, ECS was not an independent prognostic factor for OS (HR = 1.17; 95% CI, 0.87–1.57; *P* = 0.299; Figure 3B).

**FIGURE 4:**
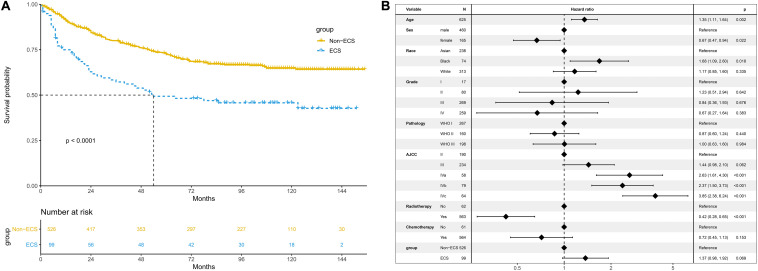
Prognosis of ECS for CSS in the unmatched cohort. **(A)** CSS between the ECS and non-ECS groups. **(B)** Multivariable regression analysis of prognostic factors for CSS. ECS, extracapsular spread; CSS, cancer-specific survival.

The non-ECS group showed a more favorable 10-year CSS than the ECS group (64.8 vs. 45.7%, *P* < 0.001; Figure 4A). However, ECS was not an independent risk prognostic factor for CSS (HR = 1.37; 95% CI, 0.98–1.92; *P* = 0.069; Figure 4B).

### Survival After PSM

**FIGURE 5:**
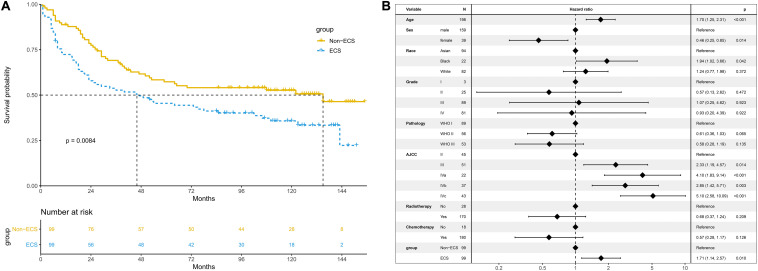
Prognosis of ECS for OS in the propensity-matched cohort. **(A)** OS between the ECS and non-ECS groups. **(B)** Multivariable regression analysis of prognostic factors for OS. ECS, extracapsular spread; OS, overall survival.

After PSM, 99 patients with non-ECS and 99 patients with ECS were matched. In the matched cohort, the ECS group showed a worse 10-year OS than the non-ECS group (35.8 vs. 52.7%; *P* = 0.008; Figure 5A). In the multivariable regression analysis, ECS was an independent risk prognostic factor for OS (HR = 1.71; 95% CI, 1.14–2.57; *P* = 0.010; Figure 5B).

**FIGURE 6:**
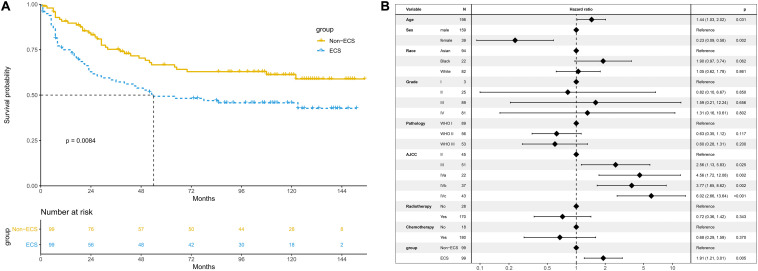
Prognosis of ECS for CSS in the propensity-matched cohort. **(A)** CSS between the ECS and non-ECS groups. **(B)** Multivariable regression analysis of prognostic factors for CSS. ECS, extracapsular spread; CSS, cancer-specific survival.

The 10-year CSS of the ECS group was worse than that of the non-ECS group (45.7 vs. 61.2%; *P* = 0.008; Figure 6A). In the multivariable regression analysis, ECS was an independent risk prognostic factor for CSS (HR = 1.91; 95% CI, 1.21–3.01; *P* = 0.005; Figure 6B).

## Discussion

This study revealed that patients with NPC with ECS of cervical lymph node metastases had worse OS and CSS compared with patients without ECS. ECS was a poor prognostic factor of NPC. The results indicated that the presence of ECS reflected aggressive biological behavior of NPC. Patients with ECS of cervical lymph node metastases might require more intensive treatments to achieve better survival.

Extracapsular spread was not included in the 8th edition of the AJCC staging system for two reasons (16). First, the detection accuracy of ECS should be improved. The gold standard of diagnosis for ECS is pathology. However, the ECS of cervical lymph node metastases in NPC is mainly assessed by image information, which is unable to reflect the true ECS status of cervical lymph node metastases detected by pathology. Previous studies focused on the overall accuracy of magnetic resonance imaging for detecting ECS revealed a specificity of 72–78%, a sensitivity of 74–80%, and an accuracy of 76–86% (17, 18). On the other hand, ECS in NPC is subjective and might lead to a wider variation in interpretation. Interobserver agreement showed substantial difference in the identification of ECS, varying from 33.6 to 75.6% among studies (4–6).

Second, several studies have suggested that ECS is not a prognostic factor. Guo et al. (6) found that no significant prognostic values for ECS were found in terms of distant metastasis-free survival (*P* = 0.264), regional relapse-free survival (*P* = 0.931), and OS (*P* = 0.629). Similarly, Li et al. (5) revealed that regional failure (HR = 1.41; 95% CI, 0.47–4.22; *P* = 0.54), distant failure (HR = 1.27; 95% CI, 0.83–1.95; *P* = 0.26), and disease failure (HR = 1.35; 95% CI, 0.94–1.95; *P* = 0.11) were not different between patients with ECS and patients without ECS.

In contrast, Mao et al. (4) reported that ECS was an independent prognostic factor for distant failure (*P* < 0.01) and disease failure (*P* < 0.01). Similarly, the current study also revealed that ECS was associated with a poor OS and CSS. The different results between these studies might be attributed to the different radiotherapy techniques used. In the study of Mao et al. (4), 83.7% of patients were treated with two-dimensional conventional radiotherapy (2D-CRT), 12.7% of patients were treated with intensity-modulated radiation therapy (IMRT), and 3.6% of patients received three-dimensional radiotherapy. In contrast, the patients in the other two studies were all treated with IMRT. It was reported that IMRT improves survival compared with 2D-CRT (19, 20). Thus, it is possible that patients with ECS receiving IMRT might achieve similar survival as patients without ECS (5, 6).

However, the efficacy of radiotherapy on survival is not well identified for patients with NPC. Although IMRT was reported to be superior to 2D-CRT (19, 20), several studies have revealed that no difference was observed in OS between IMRT and 2D-CRT (21, 22). Given the limitations of the SEER database, the radiotherapy technique could not be extracted in this study. Thus, we could not assess the impact of radiotherapy techniques on survival. Further studies should be performed to verify the results in the era of IMRT.

Extracapsular spread might be a factor that indicates aggressive biological behavior. The logistic regression analysis in the current study revealed that ECS was only associated with AJCC stage. Compared with that of non-ECS, the incidence of ECS was higher in stages IVb and IVc diseases. Similarly, Mao et al. (4) reported that nodal size was statistically correlated with ECS (*P* < 0.001). ECS was more likely to present in large lymph nodes. These results might suggest that NPC tumor cells rapidly metastasize into cervical lymph nodes, which leads to ECS. Then, these tumor cells could metastasize to distant locations.

Pathology of cervical lymph node metastases in NPC is not available in clinical practice. The assessment of ECS is mainly based on physical examination and imaging instead of pathology. Lymph nodes with ECS might invade the skin, adjacent soft tissues, or nerves. These signs could be found by physical examination. In imaging, lymph nodes presenting indistinct margins, irregular nodal capsular enhancement, infiltration into the adjacent tissues, or fusion status could be diagnosed as ECS (11–13). Moreover, no significant difference of sensitivity (*P* = 0.1317) and specificity (*P* = 0.3173) for the identification of ECS using MRI and CT were observed (17). In the SEER database, methods of ECS assessment could not be extracted. As a result, this study could not identify the sample sizes of ECS diagnosed with MRI, CT scan, or physical examination. This limitation might influence the OS and CSS in this study. On the other hand, prognostic value of ECS based on imaging in patients with NPC was inconsistent between previous studies (4–6, 9). Thus, further studies with large sample sizes are needed to verify the prognosis of ECS in NPC.

This study had some limitations. First, data on local-regional failure and distant failure were not available due to the limitations of the SEER database. The impact of ECS on local-regional-free survival and distant metastasis-free survival could not be assessed. It is important to develop treatments for patients with ECS in clinical practice. Second, selection biases inherently existed in this retrospective cohort study, which made the intrinsic quality of the data poor. To control potential biases, we performed several analytic techniques, including multivariable adjustment and PSM. The multivariable analysis in the matched cohort revealed that ECS was an independent risk factor for OS and CSS.

## Conclusion

This study indicated that ECS is a prognostic risk factor of NPC. Due to the limitations of the SEER database and retrospective nature of this study, the results should be treated with caution.

## Data Availability Statement

The datasets generated for this study are available on request to the corresponding author.

## Author Contributions

X-BP contributed to the conception of the study. XY and LL performed the data analyses, contributed to manuscript preparation, and helped to perform the analysis with constructive discussions. All authors contributed to the article and approved the submitted version.

## Conflict of Interest

The authors declare that the research was conducted in the absence of any commercial or financial relationships that could be construed as a potential conflict of interest.
